# Dissecting dysfunctional crosstalk pathways regulated by miRNAs during glioma progression

**DOI:** 10.18632/oncotarget.8265

**Published:** 2016-03-22

**Authors:** Yunpeng Zhang, Yanjun Xu, Feng Li, Xiang Li, Li Feng, Xinrui Shi, Lihua Wang, Xia Li

**Affiliations:** ^1^ College of Bioinformatics Science and Technology, Harbin Medical University, Harbin 150081, China; ^2^ Department of Neurology, The Second Affiliated Hospital, Harbin Medical University, Harbin 150081, China

**Keywords:** miRNAs, pathway crosstalk, glioma progression

## Abstract

Glioma is a malignant nervous system tumor with a high fatality rate and poor prognosis. MicroRNAs (miRNAs) are important post-transcriptional modulators of glioma initiation and progression. Tumor progression often results from dysfunctional co-operation between pathways regulated by miRNAs. We therefore constructed a glioma progression-related miRNA-pathway crosstalk network that not only revealed some key miRNA-pathway patterns, but also helped characterize the functional roles of miRNAs during glioma progression. Our data indicate that crosstalk between cell cycle and p53 pathways is associated with grade II to grade III progression, while cell communications-related pathways involving regulation of actin cytoskeleton and adherens junctions are associated with grade IV glioblastoma progression. Furthermore, miRNAs and their crosstalk pathways may be useful for stratifying glioma and glioblastoma patients into groups with short or long survival times. Our data indicate that a combination of miRNA and pathway crosstalk information can be used for survival prediction.

## INTRODUCTION

Glioma is a common malignant nervous system tumor. It can be classified as different malignant progression subtypes, including grade II, grade III and grade IV glioblastoma (GBM), which is characterized by a poor prognosis [1–3]. Although a great effort has been made to study the molecular mechanisms of glioma initiation and progression, currently the high fatality rate of this disease is still unchanged. Oncogenesis and tumor progression are complex processes [4], which are dominated by co-operation of multiple modulators and biological processes. To elucidate the mechanisms underlying glioma progression, it is important to analyze the molecular regulation and dysfunctional biological function in the malignant progression.

MicroRNAs are small non-coding RNAs, which reversely regulate coding gene expression by targeting the seed regions of target genes [5, 6]. It has been well reported that miRNAs regulate many key processes involved in tumor progression, such as proliferation, viability, migration, and invasiveness of tumor cells [6, 7]. Many recent studies have focused on identifying the signature miRNAs of glioma. *Malzkorn et al.* identified 12 up-regulates and 2 down-regulated miRNAs during glioma progression from low grade to high grade glioma, based on the miRNA expression profiles [8]. Further validation experiments have indicated that miR-17 and miR-184 are two critical regulators in the glioma progression. The study of *Ma et al.* has suggested that reduced expression of miR-544 is closely related with glioma, and has nominated miR-544 as a novel biomarker of malignant progression [9]. In addition, *Moller et al.* have reviewed more than 200 miRNAs that modulate the hallmark processes of initiation and progression of glioma [10]. However, the specific mechanisms of how these miRNAs regulate the glioma malignant progression are still unclear.

Recently, the emerging miRNA-target databases and the integration of miRNA-mRNA expression strategy have provided opportunity to investigate the miRNAs functions through examining their target genes in specific pathways. Analyzing miRNA-regulated functional pathways can shed new light on the underlying mechanisms of malignant progression of glioma. For example, Hsa-mir-7 affects the GBM invasiveness and malignant growth by targeting FAK and EGFR, which are key genes in the focal adhesion pathway [11, 12]. Moreover, a living body is a complex system; there is an inherent interdependency of biological pathways, which are defined as crosstalk of pathways [13, 14]. In order to understand the underlying mechanisms of glioma malignant progression, the dysfunctional crosstalk pathways should be analyzed.

In this study, we systematically analyzed the dysfunctional crosstalk pathways that are regulated by miRNAs during glioma malignant progression. Network analysis reveal some key miRNA-pathway collectively regulation pattern in the progression of glioma. The analysis results also suggest that miRNA regulated Cell cycle and P53 pathway play important role in the grade II to grade III progression and the cell communications related pathways may highly associated with the high grade (GBM) progression of glioma. In addition, our data indicate that a combination of miRNA and pathway crosstalk information can be used for survival prediction.

## RESULTS

### Global properties of miRNAs and genes associated with glioma malignant progression

**Figure 1:**
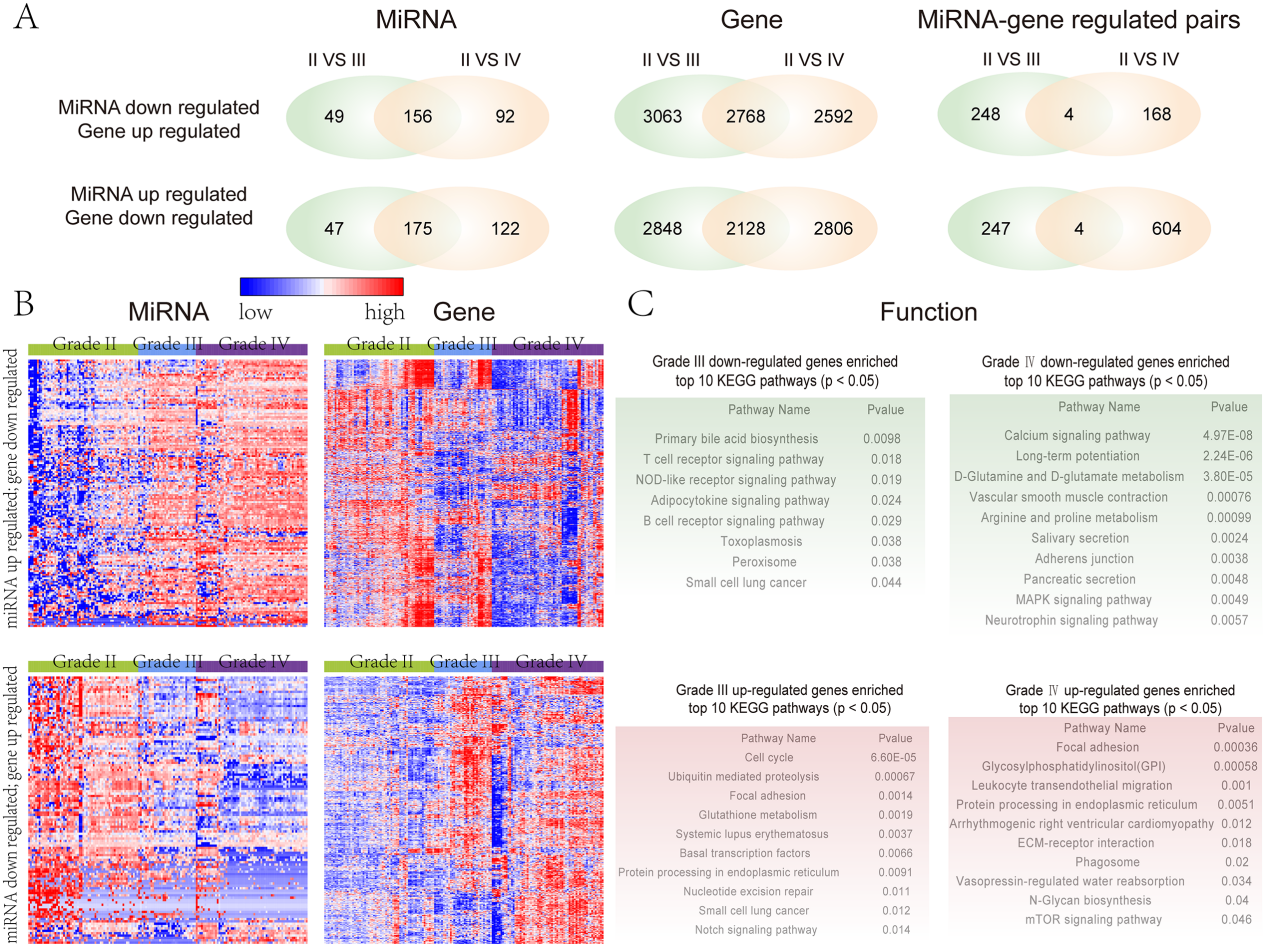
Global view of the glioma malignant progression related miRNAs and genes **A.** The left panel is the venn plot of glioma malignant progression related miRNAs by comparing low grade with high grade and GBM glioma respectively. The middle panel is the venn plot of glioma malignant progression related genes by comparing low grade with high grade and GBM glioma respectively. The right panel is the venn plot of relationships between the miRNAs and target genes that associated with the progression of glioma. **B.** The expression pattern of glioma malignant progression associated miRNAs and their target genes. The expression pattern of miRNAs and their target genes were obtained by using one-dimensional hierarchical cluster. The up (bottom) panel is the expression pattern of the up-regulated (down-regulated) miRNAs and their down-regulated (up-regulated) target genes when comparing the grade II glioma samples with grade III and grade IV glioma samples. **C.** Pathway enrichment analysis of four sets of target genes, indicating the functional roles of their regulated miRNA sets in different glioma progression stage. Red color corresponds to up-regulated genes and green color corresponds to down-regulated genes.

We identified the differential miRNAs and genes in glioma different grades (III vs IV) by using unpaired Student's t-test; all samples were compared to grade II glioma samples used as controls. In total, 222 and 297 miRNAs were up-regulated, while 4976 and 4934 genes were down-regulated in grades III and IV glioma, respectively. In contrast, there were 205 and 248 down-regulated miRNAs, and 5831 and 5360 up-regulated genes in the grade III and IV glioma, respectively (Figure 1A). These results indicate that a large scale of genes and miRNAs exhibit the expression disorder in the glioma malignant progression. In order to explore the relationship between these differential miRNAs and genes during glioma progression, we filtered the differential miRNAs and genes by the following two fundamental criteria: 1) there are computational target prediction relationships between these differential miRNAs and genes; 2) the expression of differential miRNAs and target genes is inversely correlated. We set the inverse correlated cut-off as -0.4 (P<0.05). We defined these retained miRNAs and genes as signature miRNAs and genes that are associated with the malignant progression of glioma. In the miRNA down- and target gene up-regulated group, 113 miRNAs and 364 target genes were retained comprising 252 and 172 miRNA-target gene pairs in the grade III and IV glioma respectively. For the miRNA up- and target gene down-regulated group, there were in total 151 miRNAs and 657 target genes comprising 251 and 608 miRNA-target gene pairs, respectively (Figure 1A). In both relation groups, there are only 4 overlap miRNA and target gene pairs between glioma grades III and IV. This suggests that miRNAs play diverse roles during the glioma malignant progression, via regulating different target genes in a particular stage of glioma progression. The overlap of miRNA-target pairs in both groups mainly included miRNAs, such as hsa-miR-124, hsa-miR-383, and hsa-miR-139-3p. Especially, in the miRNA down-regulated overlap group, hsa-miR-139-3p target SCARF2 pair is included and hsa-miR-139-3p is consistently down-regulated in the glioma grades III and IV. It has been previously shown that down-regulated hsa-miR-139-3p is associated with glioma progression [10]. This may suggest that hsa-miR-139-3p is a repressor of glioma malignant progression and its up-regulated target gene SCARF2 may be a risk gene for glioma.

We then visualized the expression pattern of these selected differential miRNAs and target genes across different grades of glioma by using hierarchical clustering (see methods). From the global view, the differential miRNAs are consistently up-regulated (down-regulated) in the different grades of glioma malignant progression, while their target genes are consistently down-regulated (up-regulated) (Figure 1B). We further investigated the function of these differential miRNAs during glioma malignant progression via these inversely correlated target genes by using pathway enrichment analysis. The pathway enrichment analysis was carried out respectively for the up-regulated and down-regulated genes of grades III and IV. The results suggest that these miRNAs-regulated pathways involve cancer-related pathways, such as ‘MAPK signaling pathway’, ‘adherens junction’, ‘calcium signaling pathway’, ‘focal adhesion’ and ‘mTOR signaling pathway’ (Figure 1C). For example, the ‘focal adhesion’ pathway is significantly up-regulated in grades III and IV glioma (Figure 1C). The ‘focal adhesion’ pathway has been reported to be closely associated with glioma or cancer [30]. In this context, the focal adhesion kinase (FAK) was shown to mediate tumor cell migration and tumor invasion [31]. *Liu et al.* have demonstrated that inhibition of FAK activity suppresses glioma proliferation [32]. The ‘focal adhesion’ pathway is consistently activated during glioma malignant progression as it is enriched by up-regulated target genes in glioma grades III and IV.

By dissecting miRNAs and genes that are associated with glioma malignant progression, we found that signature miRNAs play diverse roles in the glioma malignant progression via regulating different target genes during different stages of glioma progression. Furthermore, functional analysis suggests that these target genes that are regulated by signature miRNAs participate in many hallmark pathways of cancer, indicating that these miRNAs play critical roles in the initiation and progression of glioma.

### Dissecting miRNA-pathway crosstalk network implicated in the malignant progression of glioma

**Figure 2:**
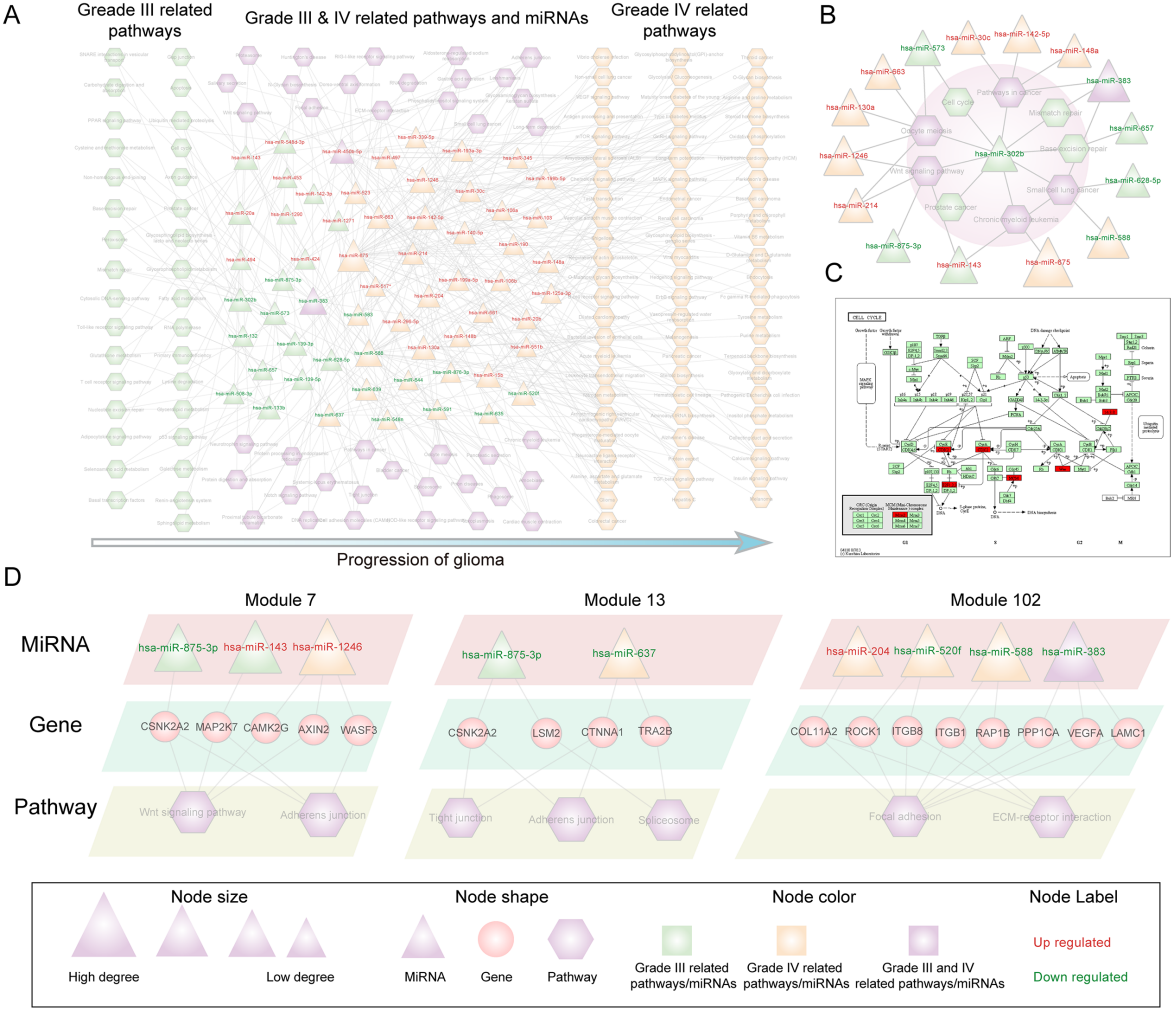
The global miRNA-pathway crosstalk network and several modules The triangle and rectangles in the network correspond to miRNAs and pathways respectively. **A.** Glioma malignant progression associated miRNA-pathway network. A miRNA and pathway connected if the target genes of miRNA were enriched in the pathway under the significance level Pvalue <0.05. **B.** Hsa-miR-302b centered module of the glioma malignant progression associated miRNA-pathway crosstalk network. **C.** Cell cycle pathway that regulated by the glioma progression related miRNAs, in which the target genes of hsa-miR-302b were annotated. Target genes were marked with red node. **D.** Three modules (module 7, 13 and 102) of the miRNA-pathway crosstalk network, in which the miRNA target genes of the corresponding pathway were shown.

For each glioma-associated miRNA, we first identified its crosstalk pathways via its inversely correlated target genes (see Methods). The significant miRNA-pathway relationships (P < 0.05) were retained to construct the glioma malignant progression associated miRNA-pathway crosstalk network. In total, 21 down-regulated miRNAs, 39 up-regulated miRNAs, and 142 KEGG pathways are involved in the network (Figure 2A). Among these KEGG 142 pathways, 33 pathways are uniquely associated with grade III glioma, while up to 70 pathways are uniquely associated with grade IV glioma; 39 pathways are associated with both grades of glioma. These data suggest that there are broader dysregulation biological pathways in the high-grade (grade IV) glioma. Moreover, there are many glioma and tumor related miRNA-regulated pathways in the networks, such as ‘glioma’, ‘apoptosis’, ‘Wnt signaling pathway’, ‘MAPK signaling pathway’, ‘cell cycle’, ‘focal adhesion’, ‘p53 signaling pathway’ and ‘mTOR signaling pathway’.

Then, we focused on the hub miRNAs as they affect most of the pathways in the whole crosstalk network and are especially important for the stability of the biological network. We defined the hub miRNAs as the top 15% of miRNAs by degree. In the network, there are 9 hub miRNAs including 6 up-regulated miRNAs (hsa-miR-15b, hsa-miR-130a, hsa-miR-142-5p, hsa-miR-1246, hsa-miR-675 and hsa-miR-143) and 3 down-regulated miRNAs (hsa-miR-520f, hsa-miR-588 and hsa-miR-383). The degree of these hub miRNAs ranges from 11 to 31; in total, they regulate up to 90 of the 142 pathways in the network, suggesting that these miRNAs control a broad range of functions and play important roles in the glioma progression. Most of these miRNAs including hsa-miR-15b, hsa-miR-130a, hsa-miR-142-5p, hsa-miR-143, hsa-miR-383 and hsa-miR-588 have been reported to associate with glioma [10]. Hub miRNAs, such as hsa-miR-143, mainly regulate the grade III related pathways, indicating their important role during grade II to grade III glioma progression. In contrast, hub miRNAs, such as hsa-miR-588 and hsa-miR-383, regulate grade IV-related pathways and pathways associated with both grade III and IV, respectively, indicating that these miRNAs cooperatively regulate multiple functional pathways during glioma malignant progression.

Network module analysis can provide detailed information about the miRNA regulatory mechanisms during glioma malignant progression. We identified miRNA-pathway crosstalk biclique modules using an algorithm (Materials and Methods). In total, 170 miRNA-pathway modules were detected. To exemplify how these modules can provide insight into the progression of glioma, the hsa-miR-302b centered module 25 comprising hsa-miR-302b and nine pathways was examined (Figure 2B, shadow region). In this module, hsa-miR-302b regulated 9 pathways including 4 pathways, such as cell cycle uniquely associated with grade III glioma and 5 pathways such as Wnt signaling pathway that related with both grade III and grade IV glioma (Figure 2B). These data indicate that the grade III related miRNA, hsa-miR-302b, also regulates pathways that play important role in the progression of grade IV glioma. It has been previously reported that hsa-miR-302b is associated with the glioma progression [10]. Five targets of hsa-miR-302b are involved in cell cycle pathway and they all annotated to the downstream of this pathway (Figure 2C) [33]. The glioma associated hsa-miR-302b target genes are distributed throughout the cell cycle from G1 phase to M phase (Figure 2C). It indicates that hsa-miR-302b via its target genes regulates the cell cycle pathway and is thus associated with the glioma progression. To further dissect the miRNA regulation pattern in the glioma progression, we analyzed miRNAs that regulate these 9 pathways in the module (Figure 2B). We found that many cancer hallmark pathways were cooperatively regulated by different miRNAs in the different progression stage. For example, Wnt signaling pathway is regulated by three miRNAs (hsa-miR-302b, hsa-miR-143, and hsa-miR-875-3p) in the grade III glioma, while it is simultaneously regulated by hsa-miR-214 and hsa-miR-1246 in the grade IV glioma. These results suggest that multiple miRNAs target a particular pathway in an alternative combination manner to regulate the glioma malignant progression.

We have then further dissected the miRNA-pathway crosstalk, mainly focusing on three modules, including module 7, module 13 and module102. Module 7 includes three miRNAs, two of which are associated with grade III glioma and the other one is related with grade IV glioma. Moreover, both up- and down-regulated miRNAs are contained in this module (Figure 2D). The two grade III related miRNAs (hsa-miR-143 and hsa-miR-875-3p) regulated Wnt signaling pathway and adherens junction pathway through targeting shared genes, including CSNK2A2 and MAP2K7. The grade IV related miRNA (hsa-miR-1246) regulated Wnt signaling pathway and adherens junction pathway through three different genes (AXIN2, WASF3 and CAMK) (Figure 2D). These data indicate that in different progression stages of glioma, the crosstalk between miRNAs and pathways may be different. In module 13, two down-regulated miRNAs (hsa-miR-875-3p and hsa-miR-637) were included and they related with different progression stages. These two miRNAs collectively regulated pathway shared genes in different progression stage and thus simultaneously mediated two different biological pathway (tight junction and adherens junction). Module 102 is associated with grade IV glioma and two cell communication pathways, focal adhesion and ECM-receptor interaction. MiRNAs regulated the two pathways through targeting 8 genes (Figure 2D). Interestingly, these 8 genes belong to focal adhesion pathway and miRNAs regulated ECM-receptor interaction pathway, indicating that miRNAs can also mediate the crosstalk between pathways in the progression of glioma.

### Dissecting crosstalk of dysfunctional pathways regulated by miRNAs in glioma progression

**Figure 3:**
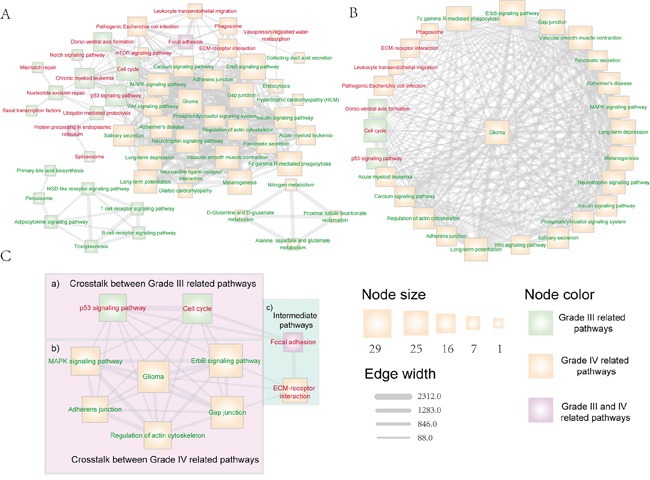
The dysfunctional pathway crosstalk network that associated with glioma malignant progression Pathways that enriched by up-regulated (down-regulated) target genes were marked with red (green) node label. Pathways that uniquely related with grade III (IV) glioma were marked with green (yellow) node fill color, while pathways that related with both grade III and grade IV glioma were marked with purple node fill color. Edge-line width is proportional to the dysfunctional crosstalk score of two pathways. Node size is proportional to their degree in the overall dysfunctional pathway crosstalk network. **A.** The overall dysfunctional pathway crosstalk network that associated with glioma malignant progression. **B.** Dysfunctional pathways that directly crosstalk with the glioma pathway. **C.** The core dysfunctional crosstalk module that related with glioma malignant progression, in which two pathway crosstalk routes were included to promote the progression of glioma: (i) direct pathway crosstalk within module between grade III and grade IV related pathways (region a&b); (ii) indirect interactions through pathways not included in module (region c).

The malignant progression of glioma is a complex process and there are many biological pathways participate in this progress. In this section, we focus on the investigation of the crosstalk between pathways that are associated with the glioma progression to understand the glioma malignant progression mechanism. We used the union set of differential and reverse expression target genes of differential miRNAs of grade III and grade IV glioma to perform pathway analysis respectively. Furthermore, the target gene sets of different grades-related miRNAs were divided into up-regulated and down-regulated groups. Then, the pathway analysis was performed independently on each target gene group. We calculated the crosstalk weight between each pathway pair of the identified pathway set and then evaluated the significance of the crosstalk weight of pathways (see methods). We selected the pathway crosstalk relationships with the significance p-value < 0.05 to construct the pathway crosstalk network of glioma malignant progression. There are 54 pathways and 335 crosstalk relationships in the network (Figure 3A). Most of the pathways in the network are known to play important roles in the initiation and progression of glioma or tumors, such as glioma, cell cycle, p53, MAPK, Wnt, ErbB and Notch signaling pathway. Pathways such as focal adhesion, regulation of actin cytoskeleton, and adherens junction participate in the communication process between cells and are regarded as the regulators of progression and invasion of malignant phenotype [34].

We first inspected the hub pathways. There are 9 hub pathways, such as ErbB, Gap junction, glioma and neurotrophin signaling pathways. In the glioma network, there are up to 26 pathways directly connected with this pathway (Figure 3B), indicating that many biological pathways regulate glioma progression. For example, the calcium signaling pathway is regarded as a critical pathway that is implicated in the progression of glioma. Calcium signaling is a ubiquitous signal transmission between cell environment and cytoplasm, and studies indicate that it is a key modulator of glioma physiology [35, 36].

We then used the CFinder algorithm to identify pathway crosstalk modules [29]. In total, 17 cliques were identified. To further dissect how pathway crosstalk module can provide insight into the progression of glioma, one glioma pathway centered module was detected, in which the pathways are highly associated with the progression of tumor (Figure 3C). In the low grade (grade II) to high grade (grade III) malignant progression of glioma, two critical pathways participate in this process, including cell cycle and p53 signaling pathway (Figure 3C, region a). In this process, cell cycle disorder and p53 signaling play important roles in the malignant progression of glioma from grade II to grade III. p53 is a key regulator of glioma development and plays a critical role during glioma malignant progression [37–39]. In the progression of low grade (grade II) to glioblastoma (grade IV), MAPK, ERBB and adherens junction pathways form dense module in the network (Figure 3C, region b). These pathways participate in the communication between cells and have been regarded as the key signatures in the malignant progression of tumor [40]. As shown in Figure 3C (region a&b), the grade III and grade IV related pathways can directly crosstalk with each other within the module. We then dissected the whole pathway crosstalk network and found that the grade III and grade IV pathways in this module can also crosstalk through another route including the Focal adhesion and ECM-receptor interaction pathway (Figure 3C, region c). In this crosstalk route, the grade III related pathways, cell cycle and p53, impact the focal adhesion pathway, which is both the grade III and grade IV related pathway. The focal adhesion pathway interacts with the ECM-receptor interaction pathway, which is a grade IV related pathway and can also crosstalk with other grade IV related pathways in the module (Figure 3C, region b). *Liu et al.* have shown that inhibition of FAK, which is a center kinase in the focal adhesion pathway, can suppress proliferation of glioma, both *in vitro* and *in vivo* [32], indicating that this route is critical for the malignant progression of glioma.

### Glioma progression associated dysfunctional crosstalk pathways and miRNAs for predicting survival in glioma

**Figure 4:**
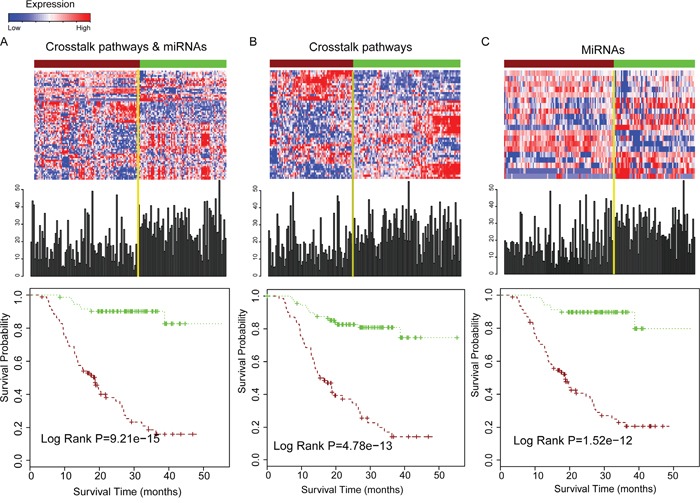
Combination of pathway crosstalk information and miRNA can gain the power for predicting survival of glioma patients **A.** Combination of crosstalk pathways of core module in Figure 3C and miRNAs for prediction the survival of overall glioma patients. The upper panel is the expression pattern of genes and miRNAs for predicting survival and the survival time distribution of all the glioma samples. The bottom panel is the estimates of survival of glioma samples based on signature genes of pathways that involved in the core module in Figure 3C and miRNAs that regulated them. **B.** Only signature genes involved in the crosstalk pathway module used for estimating patient survival. **C.** Only signature miRNAs which regulated these crosstalk pathways used for estimating patient survival.

The core miRNA regulated pathways that were contained in the dysfunctional crosstalk module were reported to play critical role in the malignant progression of glioma. We examined whether the combination of pathway crosstalk information and related miRNAs could successfully stratify the survival of glioma patients. First, we used signature genes involved in the 10 crosstalk pathways of the core module in Figure 3C and signature miRNAs that regulated these pathways to perform the survival analysis. In total, 51 signature genes and 20 miRNAs were selected. We performed K-means cluster based on the expression values of signature genes and miRNAs in the glioma samples, and the samples were divided into two groups. Then, we examined whether these two groups of patients have significantly different survival times. The patients were stratified into shorter survival time and long survival time groups based on the signature genes. The P-value was 9.21e-15 (log rank test); the median survival time of the shorter survival group (88 patients) was 16.93 months and that of the long survival group (72 patients) was 28.68 months (Figure 4A). We then used the crosstalk pathways and miRNAs to predict the survival of glioma patients respectively. The P-value was significant only when used in crosstalk pathways or miRNAs, but the prediction power is lower than when using the combination of crosstalk pathways and miRNAs (Figure 4B–4C), indicating that combination of pathway crosstalk information and miRNAs can improve the predictability of glioma patient survival.

**Figure 5:**
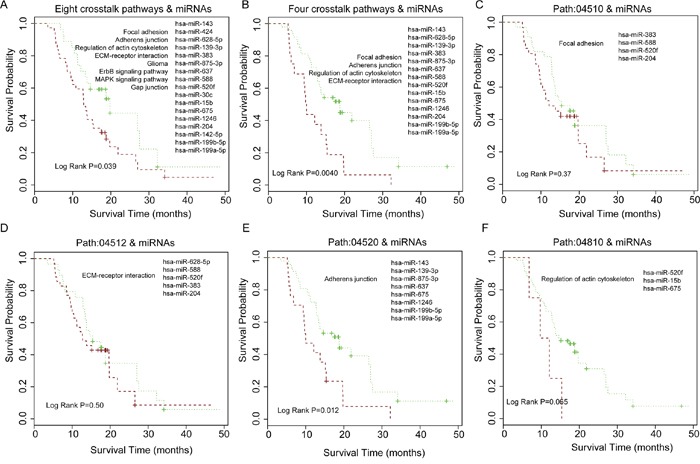
Patient survival analysis for grade IV (GBM) samples and comparison of patient survival for using pathway crosstalk information versus not **A.** Eight grade IV related crosstalk pathways in core module of Figure 3C and miRNAs regulated them for estimating GBM patient survival. **B.** Four cell communication associated crosstalk pathways in Figure 3C and miRNAs that regulated them for GBM survival analysis. **C-F.** Using only single pathway and miRNAs regulated it for GBM survival analysis.

GBM (grade IV glioma) is the most common and aggressive brain tumor, which is usually associated with poor prognosis (short survival time) [41]. We used signature genes involved in the 8 grade IV crosstalk pathways of the core module in Figure 3C and signature miRNAs that regulate them to perform the survival analysis. In total, 43 genes and 17 miRNAs were selected for the survival analysis of GBM patients. The P-value was 0.039 for the shorter survival time and long survival time group (Figure 5A). Since pathways that participate in the cell communication, such as focal adhesion are associated with malignant phenotype [34], we selected four crosstalk pathways including focal adhesion, ECM-receptor interaction, regulation of actin cytoskeleton and adherens junction, which were included in the core module and associated with grade IV glioma. In total, twenty-five genes and 14 miRNAs were used and the P-value was 0.0040 between the short and long survival group in the progression-free samples (Grade IV) (Figure 5B). We then examined whether the pathway crosstalk information can contribute to the power of survival prediction. We used only one of these four crosstalk pathways and miRNAs to predict the patient survival respectively. The results show that only one pathway and the related miRNAs could stratify the GBM patients into different survival prognosis (Figure 5C–5F), indicating that pathway crosstalk can improve survival prediction.

In summary, the combination of pathway crosstalk information and miRNAs can effectively classify both the overall glioma patients and GBM patients into short and long survival groups.

### Revalidation of combination of pathway crosstalk and miRNAs for survival prediction by TCGA independent dataset

**Figure 6:**
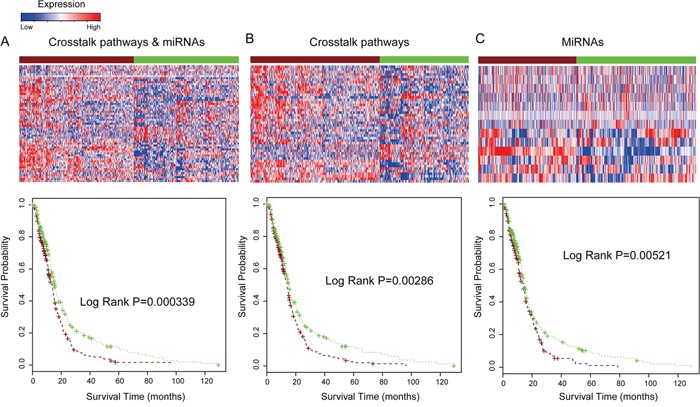
Combination of pathway crosstalk information and miRNAs predict glioma patient clinical outcome in TCGA dataset **A.** Combination of crosstalk pathways of core module in Figure 3C and miRNAs for prediction the survival of glioma patients TCGA dataset. **B.** Only signature genes involved in the crosstalk pathway module applied to predict patient survival. **C.** Only signature miRNAs which regulated these crosstalk pathways were used to predict patient survival.

To validate the prediction power of combination of pathway crosstalk information and related miRNAs, we collected independent samples with matched miRNAs and mRNA expression from TCGA database (http://tcga-data.nci.nih.gov/). For the duplicated samples, average expression values were calculated. In this study, we analyzed tumor samples from 490 glioma patients with available survival information. The gene expression in the 10 crosstalk pathways of the core module and related miRNAs were used to predict clinical outcome of samples in this TCGA independent dataset. The results show that combination of the pathway crosstalk information and related miRNAs can also stratify the survival of glioma patients into two groups with different clinical outcomes (log rank P=0.000339). The median survival time of the shorter survival group (256 patients) was 10.63 months and that of the longer survival group (234 patients) was 13.12 months (Figure 6A). We then separately applied the gene signatures involved in the core crosstalk module and miRNAs for predicting the survival of glioma samples in TCGA dataset; the P-value was 0.0029 and 0.0052 for pathway crosstalk and miRNAs, respectively (Figure 6B–6C). Consistent with the results in the original dataset, we found that the prediction power is lower than combination of pathway crosstalk information and miRNAs (Figure 6A–6C).

**Figure 7:**
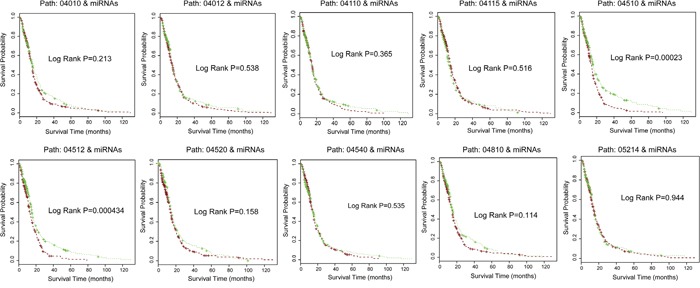
Signature genes involved in core crosstalk pathways and related miRNAs predict glioma patient clinical outcome in TCGA dataset Using only single pathway and their related miRNAs for glioma survival analysis in independent dataset. The significance of clinical outcome difference between the low-risk and high-risk groups was estimated by K-M survival analysis. P-values were calculated by the log-rank test.

We further examined the ability of pathway crosstalk information to predict clinical outcomes of glioma patients. We separately applied gene signatures that involved these 10 core crosstalk pathways and their related miRNAs to predict the survival of glioma samples in TCGA independent dataset. The results show that up to 9 of these 10 core crosstalk pathways exhibit lower power for prediction of clinical outcome of glioma patients by using only single pathway (Figure 7) compared with that of using pathway crosstalk information. One pathway (Path: 04510) has a lower p-value (log rank P= 0.00023, Figure 7) compared with pathway crosstalk information (log rank P= 0.00034, Figure 6A). Furthermore, almost all of these pathways have log rank P-value > 0.1 (Figure 7). In summary, our results show that the combination of pathway crosstalk information and related miRNAs can improve the power of prediction of clinical outcome in the independent datasets.

## DISCUSSION

MicroRNAs are important post-transcriptional modulators of the initiation and progression of glioma, one of the most common malignant nervous system tumors. Tumor progression usually results from dysfunctional co-operations of pathways that are regulated by miRNAs. In this study, to gain insight into the glioma malignant progression, we constructed the miRNA-pathway crosstalk network. Module analysis highlighted the key dysfunctional crosstalk pathways during different glioma progression stages. Our results indicate that miRNAs and their regulated crosstalk pathways in the core module can effectively stratify both glioma and GBM patients into short and long survival time groups. We also demonstrate that the combination of miRNAs and pathway crosstalk information can be used for survival prediction.

We first focused on the miRNA implicated functions in the progression of glioma. Thus, we constructed the miRNA-pathway crosstalk network, which can provide a global landscape of the miRNAs that regulate dysfunction pathways in the progression of glioma. In the miRNA-pathway crosstalk network, all miRNAs were differentially expressed and their expression inversely correlated with their target genes. The hub miRNAs, such as hsa-miR-15b, hsa-miR-130a, hsa-miR-142-5p, hsa-miR-143, hsa-miR-383, and hsa-miR-588, which regulate a wide range of dysfunctional pathways, are associated with the progression of glioma. Exploring the hsa-miR-302b centered module reveals that some key miRNAs can cooperatively regulate multiple pathways in different glioma progression stages, while multiple miRNAs target a particular pathway in an alternative combination manner to regulate the glioma malignant progression. For example, in the miRNA-pathway crosstalk module, Wnt signaling pathway is regulated by three miRNAs (hsa-miR-302b, hsa-miR-143, and hsa-miR-875-3p) in the grade III glioma, while it is simultaneously regulated by hsa-miR-214 and hsa-miR-1246 in the grade IV glioma. Moreover, the analysis of three other modules (Module7, 13 and 102) reveals that miRNAs regulated pathways share genes in different progression stages, and thus simultaneously mediate multiple biological pathways. The results indicate that our miRNA-pathway crosstalk network analysis provides a better understanding of miRNA functional regulation in the glioma progression.

The malignant progression of glioma is a complex process that involves many biological pathways. Thus, it is reasonable to investigate the dysfunctional pathway crosstalk that is implicated in the progression of glioma. In our study, the miRNA regulated pathway-pathway crosstalk network of glioma malignant progression was constructed. The crosstalk strength between each pathway pairs was evaluated by considering both the interaction strength and the dysfunction degree of genes in the two pathways. We extracted a core pathway crosstalk module implicated in the progression of glioma. Further dissection of this module revealed that miRNA regulated cell cycle and p53 pathways play important roles in the grade II to grade III progression. On the other hand, cell communications related pathways, such as focal adhesion, regulation of actin cytoskeleton, and adherens junction may be associated with the high-grade GBM progression. miRNAs and their regulated crosstalk pathways in the core module can effectively stratify both overall glioma and GBM patients into short and long survival time groups. We also show that the combination of miRNAs and pathway crosstalk information can be used for survival prediction.

In summary, our global crosstalk network analysis not only reveals some key miRNA-pathway patterns in the malignant progression of glioma, but is also helpful for charactering the functional roles of miRNAs during glioma progression.

## MATERIALS AND METHODS

### mRNA and miRNA expression profiles from Chinese Glioma Genome Atlas

The matched miRNA and mRNA expression data were downloaded from the Chinese Glioma Genome Atlas (CGGA, http://www.cgcg.org.cn/) [15, 16]; in total, 160 glioma samples were analyzed. All samples were classified according to the current WHO classification criteria of the nervous systems tumors, including 63 grade II samples, 33 grade III samples and 64 grade IV (GBM) samples.

The gene expression values were background-subtracted, quantile-normalized, and log2-transformed; the miRNA values were also log-transformed. We extracted miRNA (or gene) probes that corresponded to only one specific miRBase entry IDs (or Entrez Gene IDs) [17]. If multiple probes were associated with a common miRNA (or a gene), the expression values of these probes were averaged as the corresponding miRNA (or a gene) expression values. 818 miRNAs and 18 634 genes were included in the analysis.

### mRNA and miRNA expression profiles from the Cancer Genome Atlas (TCGA)

Independent sample matched mRNA and miRNA expression datasets of glioma were downloaded from TCGA database (http://tcga-data.nci.nih.gov/). The level 3 dataset was quantile-normalized and background-corrected for each sample in TCGA. We then calculated the average expression values for duplicate samples. In total, we analyzed the expression of 534 miRNAs and 10789 mRNAs of 490 glioma samples with available survival data.

### miRNA-target relationship data

We constructed the miRNA-target relationship by considering four major miRNA-target predicted algorithms including TargetScan [18], miRanda [17], Pictar [19] and DIANA-microT [20]. We used the union set of prediction relationship between miRNA and mRNA of these four algorithms, which were based on the previous results and consistent with the conclusion of multiple studies.

### PIP network and pathway data

We collected protein-protein interaction (PPI) network data from HPRD and STRING databases [21, 22]. HPRD is a comprehensive data source for studying the functional relationships between proteins; it includes the experimentally verified interactions and the manually curated relationships from literature. STRING database stored the protein-protein interaction data integrated from multiple data sources including both known interactions and prediction interactions. The collected data source STRING includes databases such as KEGG [23], BioGrid [24], IntAct [25], etc., and uses a combined score indicating the interaction confidence. In this study, we set a strict cut-off value (combined score >=900) to extract the high-confidence interactions. We integrated the HPRD and the high-confidence STRING interactions as the final PPI network for the subsequent analysis. We used the KEGG pathway database for the functional analysis, as it is a widely used, manually curated, and a high-quality pathway data source.

### Identifying miRNAs and genes associated with glioma malignant progression

In this study, we treated the grade II patients as control samples, and the differential miRNAs (and genes) were selected by comparing the grade III and grade IV glioma samples with the grade II samples by using the unpaired Student's t-test. We used the Benjamini and Hochberg algorithm to control the false discovery rates (FDR) [26]. The significant differential miRNAs and genes are identified with FDR < 5%.

### The hierarchical clustering

We used the hierarchical clustering algorithm, which has been implemented in the ‘gplot’ CRAN R packages to visualize the expression pattern of miRNAs and their targets in the different glioma grades. The complete linkage method was used in the hierarchical clustering. First, we clustered the samples in the same grade. Then, the samples in the same grade were ranked according to their order of the hierarchical clustering. Finally, we clustered the miRNAs (genes) by using the complete linkage method, and we analyzed their expression patterns.

### Identification of pathways that crosstalk with miRNA

To investigate the functional roles of miRNAs associated with glioma malignant progression, we used the negatively correlated targets of differential miRNAs to perform the pathway enrichment analysis and to identify pathways that crosstalk with these miRNAs. We used the cumulative hypergeometric test to evaluate the significance of each pathway that cross-talks with miRNA(s). The cumulative hypergeometric test formula can be presented as follows:

$$P=1-\sum_{k=0}^{m}\frac{\left(\begin{array}{l} n\\ k \end{array}\right)\left(\begin{array}{l} N-n\\ M-k \end{array}\right)}{\left(\begin{array}{l} N\\ M \end{array}\right)}$$ (1)

*N* represents all genome-wide genes, *M* is the number of a given pathway genes that annotated in the *N* genes, *n* is the target genes of a particular miRNA or a miRNA set, and *m* is the number of target genes in the given pathway.

### Constructing the pathways crosstalk network of glioma malignant progression

We first calculated the crosstalk weight between two pathways to evaluate their crosstalk strength in the glioma malignant progression. For two glioma progression related pathways *i* and *j*, the Fisher's method, which has previously been used by *Liu et al*., is used to calculate their crosstalk weight [27]. For differential genes that annotated in pathway *i* and *j*, we calculated their interaction strength if they have interaction (edge) in the PPI network. The crosstalk weight between two pathways is calculated as the summation of interaction strength of each interaction gene pairs. The formula of calculating interaction strength is as follows:

$$\begin{array}{lll} W(e(a,b)) & = & F(P(a),P(b),P(a,b)|\text{EX}P_{a},\text{EX}P_{b})\\ & = & -2*({\text{log}}_{e}P(a)+{\text{log}}_{e}P(b)+{\text{log}}_{e}P(a,b)) \end{array}$$ (2)

*e(a,b)* are the edges in the PPI network that is between genes in pathways *i* and *j*. *W(e(a,b))* is the interaction strength of genes *a* and *b*; *a* is the gene that is involved in pathway *i*; *b* is the gene that is involved in pathway *j*. *EXP_a_ (EXP_b_)* is the expression value of genes *a (b)* in glioma samples. *P(a) (P(b))* is the differential significance Pvalue of genes *a (b)* by using unpaired Student's t-test. *P(a,b)* is the expression correlation coefficient Pvalue between genes *a* and *b* by using the Fisher's asymptotic test.

We estimated the significance of the crosstalk strength between pathways by performing 10^6 randomization. We randomly selected the same size of genes with the number of annotated differential genes in a given pathway from the differential gene set. Then, we re-calculated the crosstalk weight between the random pathways. The frequency larger than the real crosstalk weight value is defined as the crosstalk significance Pvalue of two pathways. Finally, we extracted the interaction pathway pairs of crosstalk significance Pvalue < 0.05 and constructed the glioma malignant progression related pathway crosstalk network.

### Survival analysis

We performed survival analysis on the malignant progression related genes and miRNAs that are implicated in the hallmark pathways of glioma of the core module. First, K-mean clustering method [28] was used to classify the glioma samples into two groups based on the expression of the malignant progression related genes and miRNAs. Then, Kaplan-Meier estimate method was used to evaluate the survival difference of the two groups, and the significance was estimated by the log-rank test.

### Identification of network modules

For the miRNA-pathway regulated network, we identified biclique modules, which consist of miRNAs and their regulated pathways. A biclique module is a complete bipartite graph in which edges represent relationships between every vertex of a miRNA set to every vertex of a pathway set. The biclique modules were identified by using the algorithm downloaded from the website of the Computational Biology Laboratory in the Department of Computer Science, Iowa State University (http://genome.cs.iastate.edu/supertree/download/biclique/). For the pathway crosstalk network, we used the CFinder algorithm to identify the glioma progression related pathway crosstalk cliques [29]. A clique module is a complete graph in which edge exists between each of the pathway vertex.
